# Research progress of the DExD/H-Box RNA helicase family in T cell immune responses

**DOI:** 10.3389/fimmu.2026.2005330

**Published:** 2026-10-29

**Authors:** Wenlin Li, Kun Wu, Tiancheng Zhao, Wenbo Yang, Yongjie Zhang, Zhao Zhao, Jiankang Dong, Yujie Yang, He Li, Jianfei Wang, Weiyu Zhang, Qiang Wang

**Affiliations:** 1 Department of Urology, Peking University People’s Hospital, Beijing, China; 2 Peking University Institute of Organ Transplantation, Beijing, China; 3 Hebei North University, Zhangjiakou, China

**Keywords:** anti-tumor immunity, autoimmune diseases, DExD/H-box RNA helicases, regulatory T cells, T-cell immunity, TCR signaling, transplantation immunity

## Abstract

T-cell immunity orchestrates host defense against pathogens, tumor surveillance, and autoimmune tolerance, yet the full repertoire of post-transcriptional regulators shaping T-cell fate remains incompletely defined. The DExD/H-box RNA helicase family, best known for canonical RNA-metabolic and nucleic-acid-sensing activities, has emerged as multifaceted modulators governing multiple key steps of T-cell biology. Beyond acting as pattern-recognition sensors in antigen-presenting cells, these helicases exert cell-intrinsic control over T-cell receptor signaling, thymic development, helper-subset polarization, cytotoxic CD8^+^ T-cell effector-memory programming, and regulatory T-cell lineage commitment via chromatin remodeling, pre-mRNA splicing, translational control and transposable-element homeostasis. Accumulating pre-clinical evidence highlights their potential translational value as biomarkers or therapeutic targets, pending further validation for autoimmunity, infection, cancer and transplant rejection. Nevertheless, major gaps persist, including poorly resolved T-cell-specific functions for most helicase paralogs, obscure molecular underpinnings of their non-canonical activities, and challenges in achieving isoform-selective pharmacological targeting. Here we synthesize current mechanistic insights of DExD/H-box helicases in T-cell immunity, discuss their translational opportunities, and outline critical outstanding questions to guide future functional and therapeutic investigations.

## 1 Introduction

T cell-mediated immunity is central to adaptive immune defense, governing pathogen clearance, tumor suppression, and autoimmune pathogenesis. The activation, differentiation, and effector functions of T cells are tightly controlled by T-cell receptor (TCR) signal strength, costimulatory signals, and cytokine milieu; disruption of these regulatory networks underpins immunodeficiency, autoimmunity, and hypersensitivity. Given the clinical success of immune checkpoint inhibitors and the growing interest in regulatory T cell (Treg)-based therapies, T cell immunoregulation remains an active area of research (1–3). Importantly, T cell responses extend beyond TCR-proximal events, engaging a complex interplay of antigen presentation, downstream signaling (4), metabolic reprogramming (5), lineage specification (6), and effector execution.

DExD/H-box RNA helicases, the largest subfamily of SF2 helicases, are classified into DEAD-box and DEAH-box subgroups based on conserved amino acid motifs (7). Their hallmark catalytic core consists of two tandem RecA-like domains with at least 12 canonical conserved motifs, which recognize structural features rather than specific RNA sequences (7). In addition to this core, most members possess highly variable N- and C-terminal flanking regions that confer substrate and partner specificity, thereby endowing each helicase with distinct functional roles (8). Functionally, DEAD-box proteins primarily mediate local and transient RNA unwinding, whereas DEAH-box helicases drive processive translocation and persistent unwinding (9). These helicases participate in nearly all aspects of RNA metabolism, including transcription, splicing, ribosome biogenesis, translation, nuclear export, and RNA turnover, and their dysregulation is tightly linked to the pathogenesis of multiple human diseases (9, 10).

Emerging evidence, however, reveals that DExD/H-box helicases also exert versatile and critical immune functions beyond RNA processing. Some members act as nucleic acid sensors (11), while others broadly modulate pattern-recognition receptor pathways such as TLRs (12), RLRs (13), cGAS-STING (13), and NLRs (14). In addition, these helicases regulate T cell immunity through multiple mechanisms. DDX3Y serves as a minor histocompatibility antigen directly recognized by TCRs (15); DHX9 sustains CD8^+^ T cell survival and differentiation (16); DDX39B governs Treg lineage commitment through FOXP3 splicing (17); and helicases like DHX9, DHX36 (18), DHX15 (19), and RIG-I (20) in antigen-presenting cells sense nucleic acids to promote inflammatory cytokine production, thereby shaping Th1, Th17, or Treg polarization.

Although recent reviews have surveyed DEAD/H-box helicases in infection, innate immunity and cancer (21, 22), their T-cell-specific functions remain largely unexplored. In contrast, this review is organized around the T-cell life cycle, from thymic selection to effector, memory and Treg programs. It provides: (i) a side-by-side comparison of individual helicases across T-cell contexts; (ii) a discussion of context-dependent determinants, including cell type, differentiation stage, signaling milieu, domain usage and interaction partners; and (iii) translational implications for autoimmunity, infection, cancer and transplantation. Thus, this review complements existing broad surveys by focusing on how DExD/H-box helicases shape T-cell fate and function in a context-dependent manner.

## 2 DExD/H-box RNA helicase-mediated T cell immune regulation

DExD/H-box RNA helicases represent a core family of RNA metabolism regulators. Advances in functional studies have expanded their biological roles beyond canonical RNA processing events, including RNA splicing, ribosome biogenesis and mRNA trafficking, to a multilayered regulatory network that governs T cell immune responses. Among the 42 family members, 18 possess paralogous genes, indicating an evolutionary driving force for functional specialization (8). Such functional diversification is prominently manifested in T cell immunity, where individual members participate in multiple core processes, including T cell development (23), TCR signal transduction (16), T cell activation and proliferation (24), helper T cell subset differentiation (25), and the functional maintenance of regulatory T cells (17).

### 2.1 T cell development and thymic selection

DHX9 expression is essential for normal thymopoiesis and T cell output in thymic epithelial cells (26). DHX9 deficiency leads to a sharp reduction in the number of medullary thymic epithelial cells (mTECs), impaired proliferation and cell cycle arrest, and abnormally accelerates mTEC maturation (26). Meanwhile, DHX9 modulates thymic tuft cell differentiation by regulating Pou2f3 expression, resulting in reduced thymic output, impaired central immune tolerance and increased susceptibility to autoimmune diseases.

As a member of the DExD/H-box RNA helicase family, DDX55 is highly expressed in naive T cells and rapidly downregulated upon T cell activation. Under physiological conditions, DDX55 directly binds MYC and prevents MYC from associating with transposable elements that possess enhancer and promoter potentials. These transposable elements are adjacent to T cell activation-related genes including Cd4, Mapk3 and Rps6ka1 and contain MYC-binding motifs. DDX55 inhibits aberrant transcription of transposable elements in naive T cells to prevent the accumulation of TE-derived R-loops and genomic double-strand breaks, and maintains transposable elements in a poised state, potentially enabling rapid activation of adjacent genes following TCR stimulation (23).

### 2.2 T cell differentiation and effector regulation

DDX21 is rapidly upregulated after T cell activation, and sustains ribosome biogenesis by maintaining the expression of 47S/18S rRNA and ribosomal proteins such as RPS20 (24). It also directly binds and promotes the translation of Tfdp1 mRNA, increases TFDP1 protein abundance, induces the expression of cell cycle-related genes, facilitates G0/G1-to-S phase transition, and thereby accelerates cell cycle progression and proliferation of CD4^+^ T cells (24). T cell activation triggers the mTOR signaling pathway, which mediates DDX21 phosphorylation. This modification enhances the binding of DDX21 to AU-rich elements in the 3’ UTR of *TNF*, *IL2* and *IFN*γ mRNAs, promotes the translation of these key effector molecules, and ultimately induces robust cytokine production in T cells (27).

DHX9 directly binds the Id2 promoter via its OB_Fold domain, promotes Id2 transcription and represses E protein activity to drive the effector differentiation of KLRG1^+^IL-7R^-^ CD8^+^ T cells. In addition, the DSRM domain of DHX9 interacts with the LCK-CD3ϵ complex to ensure ZAP70 phosphorylation and ERK activation, upregulates Bcl-2 and suppresses Bim, and protects activated T cells from apoptosis (16).

In T cells, DHX36 specifically unwinds G-quadruplex structures to maintain genomic stability and negatively regulate inflammatory signaling. Under physiological conditions, DHX36 eliminates DNA G-quadruplexes in transcriptional regulatory regions and prevents TOP2-mediated DNA double-strand breaks. DHX36 deficiency leads to G-quadruplex accumulation and excessive DNA double-strand breaks. The released cytoplasmic DNA activates the cGAS-STING pathway and induces NF-κB activation, including p65 nuclear translocation and upregulation of RelB target genes, which drives the secretion of proinflammatory factors such as IL-16, IL-32, and CCL1, and sustains a persistent proinflammatory state in T cells. Clinical data show that low DHX36 expression correlates with high STING1 levels and poor prognosis in patients with B-ALL. DHX36 acts as a critical node linking G-quadruplex resolution, genomic maintenance and inflammatory repression (28). DHX36 ablation relieves the negative regulation of the cGAS-STING/NF-κB axis and promotes T cell inflammatory activation.

As a DEAH-box RNA helicase, DHX37 suppresses CD8^+^ T cell effector functions by negatively modulating the NF-κB signaling pathway. T cell activation upregulates DHX37, which localizes to the nucleolus and forms a complex with the NF-κB-associated protein PDCD11 and the p65 subunit. This complex restricts NF-κB activity and inhibits T cell activation, as well as the production of granzyme B and IFN-γ (29).

### 2.3 TCR signal transduction

RIG-I (DDX58) acts not as a signal initiator, but as a critical signal amplifier and cellular sensor. Following TCR/CD28 stimulation of naive CD4^+^ T cells, intracellular HERV-derived double-stranded RNA triggers the RIG-I pathway and activates IRF3 phosphorylation via the MAVS-TBK1 axis. For Th1 differentiation, activated IRF3 induces type I interferon production, which potentiates TCR-driven STAT1 phosphorylation, upregulates T-bet expression and facilitates Th1 cell differentiation (30). Meanwhile, activated IRF3 inhibits SMAD3 phosphorylation, blocks TGF-β-induced FOXP3 transcription, and impairs Treg differentiation and suppressive function. RIG-I deficiency indirectly suppresses Th17 generation by reducing systemic inflammatory cytokine levels. Overall, RIG-I depletion remodels TCR downstream JAK-STAT signaling, attenuates effector signal amplification, reduces pathogenic Th1 and Th17 responses, unlocks Treg functional potential, and alleviates autoimmune progression (30). Therefore, RIG-I serves as a molecular bridge linking intracellular dsRNA sensing to T cell differentiation decisions, linking innate and adaptive immunity.

### 2.4 T cell subset differentiation

DExD/H-box helicase family members exhibit distinct functional specificity during helper T cell subset differentiation. DHX9 acts as a core driver for TH17 cell differentiation. Depending on its helicase activity, DHX9 increases chromatin accessibility at the Il17a and Rorc loci, thereby facilitating the binding of canonical transcription factors including SMAD2, SMAD3, STAT3 and IRF4 to promote the transcription of TH17 signature genes. DHX9 interacts with Nono and synergistically regulates the transcriptional activity of the Il17a promoter, and the regulatory effect of Nono on Il17a is strictly dependent on DHX9. The upstream IL-6-STAT3 pathway directly binds the Dhx9 promoter to upregulate DHX9 expression, forming a positive feedback loop to amplify TH17 differentiation (25).

In RORγt^+^ Treg cells, DDX5 functions as a transcriptional corepressor that inhibits IL-10 production by restricting HIF1α expression, thereby impairing the suppressive capacity of RORγt^+^ Treg cells and promoting intestinal inflammation. DDX5 deficiency significantly enhances IL-10 secretion and contact-dependent suppressive function in RORγt^+^ Treg cells, which protects against T cell-mediated intestinal inflammatory injury (31).

DDX39B is an essential activator for FOXP3 pre-mRNA splicing. DDX39B knockdown causes prominent retention of multiple C-rich polypyrimidine tract-containing introns in FOXP3, indicating that FOXP3 splicing is highly sensitive to DDX39B abundance. DDX39B facilitates efficient splicing of C-rich polypyrimidine tract-containing introns via its ATPase activity (32). DDX39B depletion markedly reduces FOXP3 expression and abolishes the immunosuppressive properties of Treg cells. As an upstream kinase, MAP4K2 directly phosphorylates DDX39B at the Thr389 residue to trigger DDX39B nuclear translocation, thereby promoting FOXP3 splicing and Treg differentiation (17).

In Treg cells, DDX41 acts as a binding partner of FOXP3. It is preferentially enriched and rapidly upregulated during early Treg differentiation. DDX41 is dispensable for Treg generation but specifically maintains the suppressive function of mature Treg cells. DDX41 sustains negative immune regulation of effector cells by stabilizing CTLA-4 expression and modulating the CD73-adenosine metabolic pathway (33).

In summary, DExD/H-box RNA helicases constitute a comprehensive regulatory network that governs multiple T cell processes, including development, TCR signal transduction, activation and proliferation, subset differentiation, effector function execution, and migration and homing (7). Individual family members exert distinct or even opposing functions in different immune contexts. The conserved catalytic core executes fundamental RNA metabolic functions, while the variable N- and C-terminal regions confer specific protein-protein interaction capacities to adapt individual helicases to distinct steps of T cell immune responses. The functional expansion of DExD/H-box helicases from canonical RNA metabolism to precise T cell immune regulation supports their important roles in adaptive immunity (**Figure 1**).

**Figure 1:**
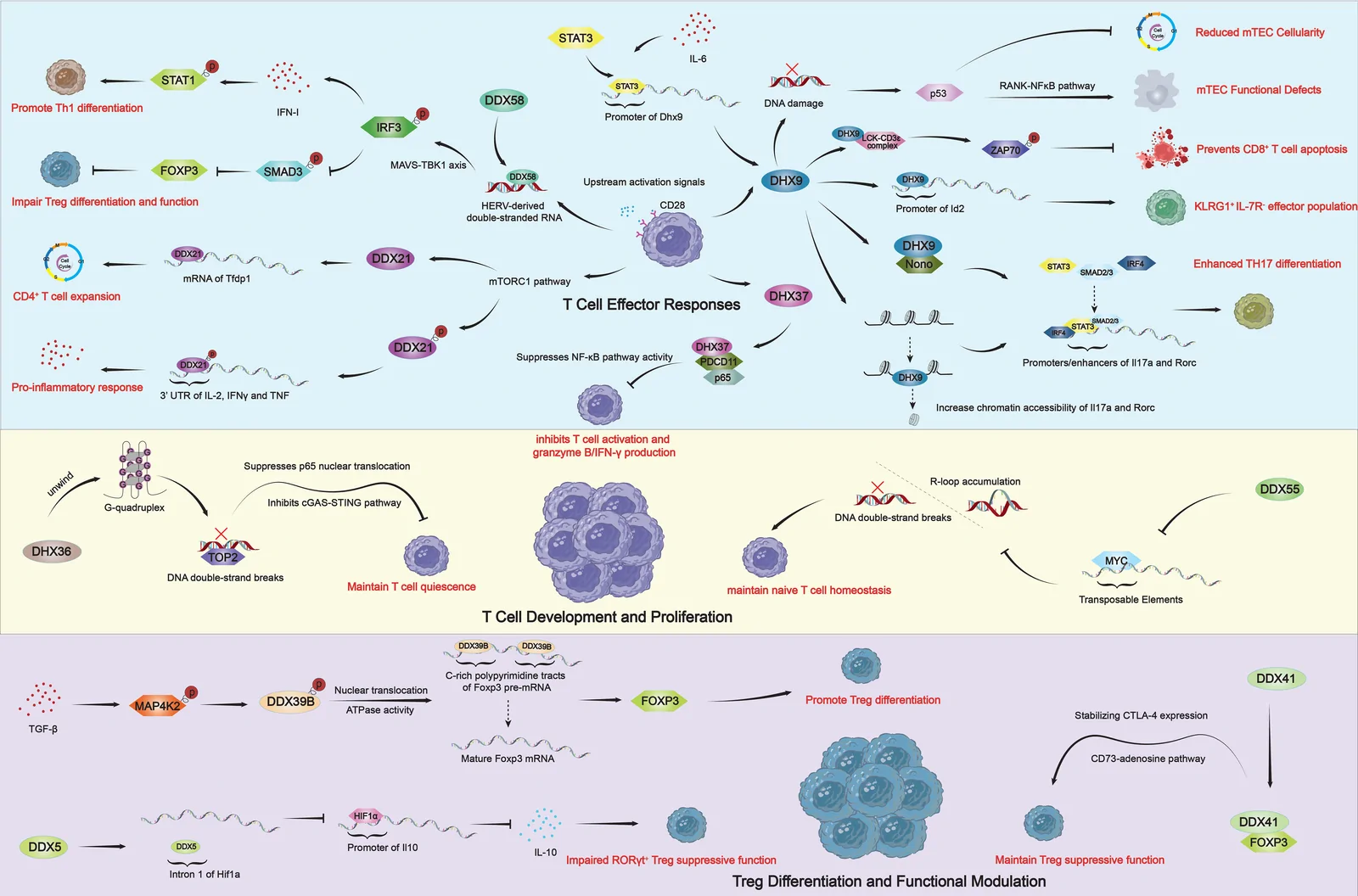
Schematic overview of DExD/H-box RNA helicase-mediated regulatory networks governing T-cell immunity. This schematic summarizes core molecular circuits by which DExD/H-box RNA helicases orchestrate multiple T-cell biological processes, including thymic development, TCR-triggered activation and proliferation, helper-T-cell subset specification, cytotoxic CD8^+^ T-cell effector responses, and regulatory T-cell (Treg) differentiation and suppressive function. Distinct helicases exert context-dependent positive or negative control over downstream transcription programs, chromatin accessibility, RNA splicing, post-transcriptional translation, nucleic-acid-sensing signaling, and transposable-element homeostasis. Solid arrows indicate promoting effects; blunt-ended lines indicate inhibitory effects. IFN-I, type I interferon; MAVS, mitochondrial antiviral signaling protein; TBK1, TANK-binding kinase 1; IRF, interferon regulatory factor; STAT, signal transducer and activator of transcription; SMAD, SMAD family member; FOXP3, forkhead box protein P3; DDX, DEAD-box helicase; DHX, DEAH-box helicase; HERV, human endogenous retrovirus; mTORC1, mammalian target of rapamycin complex 1; IL, interleukin; TNF, tumor necrosis factor; NF-κB, nuclear factor-kappa B; PDCD1, programmed cell death protein 1; ZAP70, zeta-chain-associated protein kinase 70; Nono, non-POU domain-containing octamer-binding protein; Rorc, RAR-related orphan receptor gamma; mTEC, medullary thymic epithelial cell; p53, tumor protein p53; KLRG1, killer cell lectin-like receptor G1; TOP2, DNA topoisomerase II; cGAS, cyclic GMP-AMP synthase; STING, stimulator of interferon genes; MYC, MYC proto-oncogene; TGF-β, transforming growth factor-beta; MAP4K2, mitogen-activated protein kinase kinase kinase 2; ATPase, adenosine triphosphatase; HIF1α, hypoxia-inducible factor 1α; CTLA-4, cytotoxic T-lymphocyte-associated protein 4; CD, cluster of differentiation; Treg, regulatory T cell; RORγt, RAR-related orphan receptor gamma t.

### 2.5 Context-dependent duality and determinants of helicase function

The functions of DExD/H-box helicases in T cells are not unidirectional but are dictated by cell type, differentiation stage, signaling context, and molecular partnerships. DHX9 exemplifies this duality. In CD4+ T cells, IL-6–STAT3-driven DHX9 increases chromatin accessibility at the *Il17a* and *Rorc* loci and cooperates with Nono to promote TH17 differentiation (25). In CD8+ T cells, the DSRM domain of DHX9 stabilizes LCK–CD3ϵ–ZAP70–ERK signaling and Bcl-2 expression to support survival before clonal expansion, whereas its OB_Fold domain binds the *Id2* promoter to drive KLRG1+IL-7R− effector differentiation (16). In mTECs, DHX9 is required for medullary thymic epithelial cell proliferation and thymic tuft cell differentiation, thereby sustaining central tolerance (26). Conversely, DDX5 restricts HIF1α/IL-10 expression in RORγt+ Tregs and impairs their suppressive function (31), while in CD8+ T cells it represses TCF1 and Eomes and cooperates with the Malat1–PRC2 complex to promote terminal effector/effector memory differentiation at the expense of central memory T cells (34, 35). Thus, apparently contradictory outcomes reflect differences in lineage, activation state, partner proteins, domain usage, subcellular localization, post-translational modifications, and the availability of RNA or chromatin substrates.

## 3 DExD/H-box helicases in tumor cellular immunity

DExD/H-box helicases exert complex, context-dependent dual roles in tumor immune regulation and participate in multiple critical processes of T cell activation, differentiation, cytotoxicity and exhaustion. Their functions are highly dependent on cell types and signaling contexts.

### 3.1 DHX37 regulates CD8^+^ T cell-mediated tumor immune evasion

T cell immunity is central to the elimination of malignant cells, yet its efficacy is profoundly constrained by tumor immune evasion mechanisms and the immunosuppressive tumor microenvironment. DHX37 was identified as a negative regulator of CD8^+^ T cell function through *in vivo* CRISPR screening. Mechanistically, DHX37, via its DEXDc and HELICc domains, interacts with NF-κB p65 and PDCD11 to suppress TCR-driven NF-κB pathway activation. Loss of DHX37 relieves this inhibition, leading to upregulation of the activation marker CD69 and the costimulatory receptor CD27, accompanied by robust secretion of granzyme B and IFN-γ, thereby markedly enhancing cytotoxic function. Single-cell transcriptomic analysis further revealed that Dhx37-deficient tumor-infiltrating CD8^+^ T cells upregulate genes associated with T cell activation and effector function, including Tox, Nr4a2, and Eomes. In the E0771 triple-negative breast cancer model, adoptive transfer of Dhx37-knockout CD8^+^ T cells significantly delayed tumor progression. Knockout of DHX37 in human CD8^+^ T cells similarly augmented degranulation and cytotoxicity, indicating that this regulatory mechanism is conserved between mouse and human CD8^+^ T cells (29). Collectively, these findings identify DHX37 as a negative regulator of CD8^+^ T cell effector function, suggesting that targeting this molecule may improve the efficacy of adoptive T cell therapy against solid tumors.

### 3.2 DDX39B regulates treg differentiation and immunosuppression

DDX39B is a core splicing factor governing Treg differentiation and function. Downregulation of DDX39B causes retention of multiple FOXP3 introns, particularly those containing C-rich polypyrimidine tracts, thereby preventing the production of functional FOXP3 protein and impairing Treg differentiation and suppressive capacity (36). Mechanistically, MAP4K2 kinase directly phosphorylates DDX39B at Thr389 to promote its nuclear translocation, which enhances FOXP3 mRNA splicing efficiency and drives Treg maturation. Both loss of MAP4K2 kinase activity and DDX39B ablation abrogate this process. In a pancreatic cancer model, Treg-specific deletion of Map4k2 resulted in a marked reduction of intratumoral Tregs and a concomitant increase in CD8^+^ T cell infiltration, substantially improving responsiveness to anti-PD-1 therapy (17). These findings indicate that the MAP4K2–DDX39B–FOXP3 axis modulates the tumor immune microenvironment through its effects on Treg-mediated immunosuppression.

### 3.3 RIG-I functions as an immune checkpoint restricting CD8^+^ T cell anti-tumor immunity

RIG-I (DDX58) is specifically and highly expressed in tumor-infiltrating CD8^+^ T cells, most prominently in terminally exhausted subsets, and has been identified as a novel intracellular immune checkpoint. Mechanistically, sustained TCR signaling in the tumor microenvironment drives RIG-I upregulation, which subsequently competes with AKT for binding to Src via its CARD domain, thereby inhibiting the PI3K/AKT/glycolysis axis and impairing effector differentiation and metabolic fitness of CD8^+^ T cells. RIG-I ablation reverses this exhaustion program, promotes the differentiation of central memory and effector T cell subsets, and significantly enhances the release of perforin, granzyme B, and IFN-γ. In MC38 and B16F10 tumor models, RIG-I-deficient CD8^+^ T cells exhibited superior tumor-infiltrating capacity. In B16F10 tumors, which are refractory to anti-PD-1 monotherapy, RIG-I knockout combined with anti-PD-1 blockade produced synergistic antitumor effects. Knockout of RIG-I in humanized EBV-specific CTL models similarly enhanced anti-tumor function, highlighting the translational potential of targeting RIG-I in human T cell immunotherapy (37). This study uncovers RIG-I as a metabolic checkpoint that limits the response to immunotherapy in solid tumors, suggesting that RIG-I targeting may sensitize tumors to immune checkpoint blockade.

In conclusion, DExD/H-box RNA helicases modulate multiple critical nodes in the cancer-immunity cycle, exerting context-dependent pro- or anti-tumor immune effects by mediating immune escape and immune activation. Preclinical studies suggest that targeting specific helicases may improve responses to immune checkpoint blockade. Notably, several helicases intrinsically regulate T cell differentiation and survival. Therefore, systemic targeting of these proteins requires precise balance between maximizing antitumor benefits and preserving the plasticity of immune effector cells.

## 4 DExD/H-box helicases in T cell immunity to autoimmune diseases

DExD/H-box RNA helicases exert diverse and essential functions in the regulation of T cell immunity. Their functional dysregulation is closely associated with the pathogenesis of multiple autoimmune diseases.

### 4.1 Multiple sclerosis

DDX39B is one of the most extensively investigated DExD/H-box helicase in the pathogenesis of multiple sclerosis. It acts as a core regulator of alternative splicing of IL7R. The IL7R gene encodes the α-chain of the IL-7 receptor, and exon 6 skipping of IL7R generates soluble IL7R. The multiple sclerosis risk-associated single-nucleotide polymorphism rs6897932 is located within exon 6. The risk allele enhances the activity of an exonic splicing silencer to promote exon 6 skipping. DDX39B facilitates the binding of U2AF to branch-point sequences via its ATPase activity, thereby supporting normal exon 6 splicing and repressing soluble IL7R production. Soluble IL7R enhances IL-7 bioactivity and aggravates the progression of experimental autoimmune encephalomyelitis. Genetic analyses reveal significant epistatic interactions between DDX39B and IL7R loci. Individuals homozygous for risk alleles at both loci exhibit approximately three-fold increased multiple sclerosis susceptibility (38). DDX39B also serves as an essential activator for FOXP3 pre-mRNA splicing. FOXP3 introns harbor unique cytosine-rich polypyrimidine tracts, rendering FOXP3 splicing highly sensitive to DDX39B abundance. DDX39B knockdown induces robust retention of multiple FOXP3 introns, including intron 2, 4, 6, 7, 9 and 11, leading to pronounced reductions in FOXP3 mRNA and protein levels. In CD4^+^CD25^+^ Treg cells, DDX39B deficiency disrupts the canonical Treg transcriptional signature, downregulating FOXP3 target genes such as IL2RA and upregulating GATA3, a key transcription factor for TH2 differentiation. These alterations abolish Treg suppressive function and confer an effector-like phenotype (32). DHX9 expression is upregulated in CD4^+^ T cells from experimental autoimmune encephalomyelitis mice and multiple sclerosis patients. T cell-specific *Dhx9* knockout markedly inhibits TH17 differentiation and ameliorates experimental autoimmune encephalomyelitis symptoms, indicating that multiple DExD/H-box helicases synergistically shape the T cell immune regulatory network in multiple sclerosis (25).

### 4.2 Inflammatory bowel disease

DDX21 contributes to inflammatory bowel disease pathogenesis by promoting CD4^+^ T cell proliferation at the translational level. DDX21 is highly expressed in CD4^+^ T cells from inflamed tissues, and its expression correlates closely with T cell proliferation and translational pathway activity. T cell-specific *Ddx21* ablation impairs T cell homeostasis and proliferation. In adoptive transfer colitis models, *Ddx21*-deficient CD4^+^ T cells fail to stably engraft in recipient mice and cannot induce colitis, accompanied by markedly reduced intestinal T cell infiltration. Mechanistically, DDX21 sustains global mRNA translation by supporting ribosome biogenesis during T cell activation. *Ddx21* deficiency significantly downregulates 47S/18S rRNA and ribosomal proteins such as RPS20, thereby suppressing overall translational efficiency. Polysome profiling and RNA immunoprecipitation assays demonstrate that DDX21 modulates cell cycle progression by promoting the translation of TFDP1 mRNA. Ddx21 depletion does not alter Tfdp1 transcript levels but substantially reduces TFDP1 protein abundance, leading to the downregulation of cell cycle-related genes including Mcm3–7 and Pcna. Tfdp1 overexpression partially rescues the proliferative capacity of *Ddx21*-deficient CD4^+^ T cells. In DSS-induced colitis models, Ddx21-deficient mice exhibit attenuated intestinal inflammation with reduced infiltration and proliferation of colonic CD4^+^ T cells (24). DDX5 plays a complex regulatory role in inflammatory bowel disease. DDX5 restricts the suppressive function of RORγt^+^ Tregs. DDX5 ablation relieves this restriction, enhances IL-10 production and Treg inhibitory activity, and protects against T cell-mediated intestinal inflammation (31).

### 4.3 Rheumatoid arthritis

DExD/H-box helicases participate in immune regulation in rheumatoid arthritis through T cell-intrinsic mechanisms. DHX9 expression is significantly upregulated in CD4^+^ T cells from rheumatoid arthritis patients, and T cell-specific Dhx9 knockout ameliorates clinical symptoms in collagen-induced arthritis mouse models. Mechanistically, DHX9 increases chromatin accessibility at the Il17a and Rorc loci in a helicase-dependent manner, facilitating the binding of canonical transcription factors including SMAD2, SMAD3, STAT3 and IRF4 to drive TH17 cell differentiation. The ATPase activity of DHX9 is indispensable for the transcription of Il17a and Rorc, and both helicase domain deletion and ATP-binding site mutation abolish its capacity to promote TH17 differentiation. In addition, DHX9 interacts with Nono to synergistically modulate the transcriptional activity of the Il17a promoter. The upstream IL-6-STAT3 signaling pathway directly upregulates DHX9 expression, forming a positive feedback loop to amplify TH17 differentiation (25). Collectively, these findings demonstrate that DExD/H-box helicases contribute to rheumatoid arthritis pathogenesis by modulating T cell differentiation and effector functions.

### 4.4 Systemic lupus erythematosus

Aberrant expression of DExD/H-box helicases is tightly associated with excessive activation of the type I interferon pathway in systemic lupus erythematosus. DDX5 serves as a novel serological biomarker for systemic lupus erythematosus. Serum DDX5 levels are markedly elevated in patients with systemic lupus erythematosus and positively correlate with disease activity. Combined detection of DDX5, anti-dsDNA and anti-Sm antibodies increases the area under the curve for systemic lupus erythematosus diagnosis to 0.976 (39). Genetically, the 11q23.3 locus harboring DDX6 represents a shared risk locus for systemic lupus erythematosus and Sjögren’s syndrome. Risk single-nucleotide polymorphisms within this region modulate the expression of DDX6 and adjacent genes in immune cells, indicating that genetic variants predispose to autoimmune pathogenesis by impairing the immunosuppressive function of DDX6 (40).

### 4.5 Sjögren’s syndrome

Fine-mapping analyses have identified five functional risk single-nucleotide polymorphisms within the shared DDX6-CXCR5 genetic risk locus of Sjögren’s syndrome and systemic lupus erythematosus. Three variants located between DDX6 and CXCR5, including rs4936443, rs4938572 and rs7117261, form a synergistic risk haplotype that modulates regional transcriptional activity. The variant rs4938573 resides proximal to the CXCR5 promoter. Its risk allele reduces nuclear protein binding affinity and impairs promoter activity in T cells, which is consistent with reduced CXCR5 expression in peripheral CXCR5^+^ cells from Sjögren’s syndrome patients carrying this risk haplotype. As a critical regulator of T follicular helper cell migration and function, downregulated CXCR5 disrupts T follicular helper cell trafficking to germinal centers and impairs its capacity to support B cell maturation and differentiation, thereby driving T cell-mediated autoimmune responses in Sjögren’s syndrome. Chromatin conformation capture assays reveal that this risk locus establishes an extensive regulatory network encompassing DDX6, CXCR5, PHLDB1, lnc-PHLDB1-1, BCL9L, TRAPPC4 and HINFP, suggesting that risk variants perturb T cell immune homeostasis by coordinately modulating multiple immune-related genes. Single-cell RNA sequencing analyses of minor salivary gland biopsies from Sjögren’s syndrome patients demonstrate that the allelic status of these SNPs shapes the expression patterns of multiple genes including DDX6 across distinct T cell populations (40). The functional impacts of this risk locus extend beyond canonical cis-regulation, whereby long-range chromatin looping enables coordinated modulation of multiple genes in T cells.

## 5 DExD/H-box helicases in T cell-mediated transplant rejection

T cell-mediated rejection constitutes a core barrier that restricts the long-term survival of allogeneic grafts. Disparities in minor histocompatibility antigens are sufficient to drive T cell activation and trigger graft rejection even in human leukocyte antigen-matched transplantation. The DExD/H-box helicase family has garnered extensive attention owing to its versatile functions in RNA metabolism, innate immune sensing, and adaptive immune regulation. These helicases not only serve as a protein source of minor histocompatibility antigens to directly participate in T cell allorecognition, but also critically modulate the survival, differentiation and functional programming of CD8^+^ T cells.

In HLA-matched transplantation, Y-chromosome-encoded H-Y antigens represent important targets for T-cell allorecognition. Among 15 human Y-chromosomal genes with X-chromosomal homologs, six have been verified to encode H-Y antigens, and DDX3Y (also termed DBY) is one of the most immunodominant members (44). Vogt et al. isolated the first HLA-class-II-restricted, H-Y-specific CD4^+^ CTL clone from a male patient with chronic myeloid leukemia (CML) suffering from acute graft-versus-host disease (GVHD). This clone recognizes the DDX3Y-derived 12-mer peptide (HIENFSDIDMGE) presented by HLA-DQ5, and polymorphisms at merely two amino-acid positions are sufficient to completely abrogate T-cell recognition (15). Subsequently, Rosinski et al. isolated an HLA-B*2705-restricted CD8^+^ CTL clone specific for DDX3Y_74-83_ (SRDSRGKPGY) from a male patient with acute lymphoblastic leukemia (ALL) who received a female-donor graft without GVHD. This decapeptide differs from the corresponding peptide of its X-chromosomal paralog DDX3X at four amino-acid sites (45). Notably, DDX3Y is among the few H-Y antigens that encode both MHC-class-I- and MHC-class-II-restricted epitopes, suggesting that it can concurrently activate CD4^+^ and CD8^+^ T-cell response pathways.

Following HSCT, DDX3Y/DBY not only elicits T cell responses but also induces high-titer specific antibody reactions. Miklos et al. performed ELISA screening on plasma samples from 150 HSCT patients and found that 50% of male recipients with female donors developed IgG antibodies against recombinant DBY protein, whereas only 5% of male recipients with male donors were seropositive. DBY-specific antibodies were detectable in 17% of healthy females but completely absent in healthy males. Epitope mapping revealed that these antibodies predominantly target amino acid regions divergent between DBY and DDX3X, confirming their alloantigen specificity (41). Porcheray et al. further identified persistent DBY-specific CD4^+^ T cells and serum IgG antibodies in a male MDS patient transplanted with female donor-derived grafts, which remained detectable for up to 38 months post-transplantation. The isolated CD4^+^ T cell clone (clone 42) recognizes DBY_427-444_ in an HLA-DRB1*0101-restricted manner and cross-reacts with conserved peptides from both DBY and DDX3X, while patient-derived serum antibodies strictly discriminate these two paralogs. Upon stimulation, this CD4^+^ clone secretes both Th1 (IFN-γ, TNF-α) and Th2 (IL-4, IL-10, IL-13) cytokines, exhibiting a mixed T helper phenotype (46). These findings suggest that DDX3Y/DBY elicits coordinated T and B cell responses, and DBY-specific CD4^+^ T cells provide essential helper signals for B cell-mediated humoral immunity.

The clinical relevance of DDX3Y/DBY extends beyond hematopoietic stem cell transplantation. In a retrospective study encompassing 118 renal transplant recipients, Tan et al. demonstrated that 46% (12/26) of female recipients receiving male donor kidneys developed *de novo* anti-RPS4Y1 or anti-DDX3Y antibodies after transplantation, whereas the seroconversion rate ranged only from 0% to 3% in other gender-matching groups. *De novo* H-Y antibody generation was strongly correlated with acute rejection. Among the 26 female recipients of male kidney transplants, H-Y antibody development was strongly correlated with acute rejection (P = 0.00048) and correlated with plasma cell infiltrates in biopsied kidneys (P = 0.04). Of the two H-Y antigens, RPS4Y1 was more frequently recognized (P = 0.005). Histologically, antibody-positive patients exhibited markedly increased renal infiltration of CD138^+^/CD38^+^ plasma cells (P = 0.04). No correlation was observed between H-Y antibody positivity and C4d deposition or donor-specific HLA antibodies, indicating that H-Y antibody responses were independent of canonical HLA-driven humoral rejection (42). This study provides the first clinical evidence linking H-Y antibodies to acute rejection in solid organ transplantation, establishing H-Y antigens such as DDX3Y and RPS4Y1 as clinically meaningful non-HLA immune targets in solid organ transplantation.

Active immunotherapeutic intervention targeting H-Y antigens to establish allograft tolerance has been validated in mouse models. Chen et al. employed TCR-transgenic A1×RAG1^^-^/^-^^ mice, whose TCR specifically recognizes the H-2E^k^-restricted epitope REEALHQFRSGRKPI derived from murine Dby. A series of altered peptide ligands were generated via systematic mutation of TCR contact residues. One lead variant with an arginine-to-histidine substitution at position 490 (490R→H) fails to trigger TCR internalization and delivers incomplete activating signals. Repeated *in vitro* stimulation with this variant polarizes naive T cells toward an IL-10-dominant regulatory phenotype. Female A1×RAG1^-^/^-^ mice treated with this altered peptide ligand permanently accepted male skin grafts, and the established tolerance resisted the attack of adoptively transferred naive lymphocytes, indicating successful induction of dominant transplantation tolerance. Mechanistically, tolerant mice exhibited expansion of peripheral CD4^+^CD25^+^ T cells from 0.5% to 3.0%, with an intragraft infiltration rate up to 13.0%, accompanied by elevated Foxp3 mRNA expression and partial clonal deletion of effector T cells. The total number of splenic CD4^+^ T cells decreased from 7.14×10^6^ to 2.73×10^6^. Notably, IL-10 receptor blockade failed to abrogate established tolerance, suggesting functional redundancy in this regulatory mechanism (43). This study demonstrates for the first time that altered peptide ligands designed against DExD/H-box helicase-derived minor histocompatibility antigen epitopes enable antigen-specific induction of transplantation tolerance, providing a mechanistic basis for future minor histocompatibility antigen-based tolerogenic strategies.

## 6 DExD/H-box helicases in T cell immunity to infection

### 6.1 DExD/H-box helicases in CD8^+^ T cell antiviral immunity

DHX9 represents the most well-characterized DExD/H-box helicase member governing CD8^+^ T cell antiviral immunity. Following viral infection, T cell-specific *Dhx9* knockout mice exhibit markedly impaired accumulation of antigen-specific CD8^+^ T cells, severely compromised viral clearance, and reduced survival rates. Mechanistically, DHX9 directly binds LCK via its DSRM domain to maintain the integrity of the CD3ϵ-LCK-ZAP70 signaling complex, thereby facilitating ZAP70 phosphorylation and early activation of downstream ERK signaling. *Dhx9* deficiency attenuates ERK activity, upregulates the pro-apoptotic factor Bim and downregulates the anti-apoptotic factor Bcl-2, resulting in extensive apoptosis of CD8^+^ T cells prior to clonal expansion. Ectopic Bcl-2 expression completely rescues this survival defect. In parallel, DHX9 directly interacts with the *Id2* promoter through its OB_Fold domain, sustains chromatin accessibility, and promotes *Id2* transcription to ensure proper effector differentiation. Either *Id2* overexpression or *E2A* ablation efficiently restores the defective effector differentiation of *Dhx9*-deficient CD8^+^ T cells. Collectively, DHX9 executes dual essential functions in CD8^+^ T cell antiviral immunity through distinct structural domains. The DSRM domain sustains proximal TCR signaling to support T cell survival, whereas the OB_Fold domain modulates *Id2* transcription to drive effector differentiation (16).

DDX5 serves as another well-defined DExD/H-box family member that modulates CD8^+^ T cell antiviral immunity. Louis et al. demonstrated that DDX5 positively regulates the differentiation of terminal effector cells, effector memory T cells and terminal effector memory T cells, while restraining the generation of central memory T cells. In the lymphocytic choriomeningitis virus infection model, DDX5-deficient CD8^+^ T cells exhibit a reduced frequency of KLRG1^high^ CD127^low^ terminal effector cells and an increased proportion of KLRG1^low^ CD127^high^ memory precursor cells. At the memory phase, DDX5 deficiency increases the abundance of CD62L^high^ CD127^high^ central memory T cells and decreases effector memory T cell and terminal effector memory T cell populations. Conversely, DDX5 overexpression suppresses central memory T cell formation and promotes effector lineage differentiation. Mechanistically, DDX5 drives effector lineage commitment of CD8^+^ T cells by repressing TCF1 and Eomes, two core transcription factors responsible for central memory T cell maintenance and stemness (34). Inducible DDX5 ablation in established mature memory T cells still facilitates central memory T cell retention and reduces terminal effector memory T cells, indicating that DDX5 functions not only during early T cell differentiation but also in sustaining the homeostasis of mature memory subsets. Mechanistically, DDX5 cooperates with the long non-coding RNA Malat1 to recruit the PRC2 complex for epigenetic silencing of central memory T cell-related genes, thereby fine-tuning CD8^+^ T cell fate determination. In addition, DDX5 regulates CD8^+^ T cell proliferation and survival. DDX5-deficient CD8^+^ T cells display accelerated proliferative kinetics at the early infection stage but exhibit enhanced apoptotic susceptibility (35).

### 6.2 DExD/H-box helicases in T cell priming

In antiviral immune responses, dendritic cell (DC)-resident RIG-I, MDA5, and DHX15 constitute a hierarchical regulatory network bridging RNA sensing, signal bifurcation, and T-cell priming (47). RIG-I and MDA5 recognize distinct viral RNA features and activate the mitochondrial adaptor MAVS, yet their outputs display spatiotemporal divergence: the RIG-I–MAVS axis drives early DC maturation and migration to draining lymph nodes, enabling cross-priming of naive T cells, whereas MDA5 serves as a complementary source of type I interferon (IFN-I) under conditions such as long dsRNA or LCMV infection (48, 49). The IFN-I produced by both pathways not only reinforces DC antigen processing and cross-presentation in an autocrine manner, but also shapes CD8^+^ T-cell expansion and polyfunctional differentiation through upregulation of costimulatory molecules and IL-15 (50).

DHX15 acts downstream of MAVS to selectively amplify NF-κB and MAPK signaling—independent of IRF3 phosphorylation—thereby enhancing DC secretion of proinflammatory cytokines such as IL-6 and TNF-α, which provide the third signal for T-cell polarization (47). Thus, RIG-I, MDA5, and DHX15 form an integrated axis from viral recognition and signal diversion to cross-presentation and inflammatory niche remodeling, collectively contributing to CD8^+^ T-cell survival, effector differentiation, and memory formation during RNA virus infection.

In contrast, evidence regarding the regulatory roles of DExD/H-box helicases in T cell-mediated antibacterial immunity remains limited and predominantly relies on clinical observations. Clinical studies have shown that DDX3X mRNA expression is significantly upregulated in peripheral T cells from septic patients, and its level positively correlates with disease severity and mortality (51). In animal models, DDX3X inhibition markedly restores CD4^+^ T cell abundance and prolongs survival in septic mice (52). These findings suggest that DDX3X critically modulates T cell pyroptosis during bacterial infection, although the detailed molecular mechanisms and causal relationships require further elucidation (**Table 1**).

**Table 1:** Summary of DExD/H-box helicase members described in this review.

DExD/H member	Disease/ Context	Mechanistic category	Key mechanism	Effect on T cell immunity	Evidence source	References
DDX39B	Pancreatic cancer	Anti-tumor immunity	MAP4K2 phosphorylates DDX39B at Thr389 to promote its nuclear translocation, enhancing FOXP3 splicing and Treg maturation	Promotes Treg maturation	Cell + Animal	(17)
DDX58 (RIG-I)	Melanoma	Anti-tumor immunity	Sustained TCR signaling in the tumor microenvironment upregulates RIG-I in tumor-infiltrating CD8^+^ T cells; RIG-I competes with AKT for Src binding via its CARD domain, inhibiting the PI3K/AKT/glycolysis axis and restraining effector differentiation and metabolic fitness	Attenuates antitumor effector function and anti-PD-1 efficacy	Cell + Animal	(37)
DHX37	Triple-negative breast cancer	Anti-tumor immunity	Via its DEXDc and HELICc domains, interacts with NF-κB p65 and PDCD11 to suppress TCR-driven NF-κB activation; knockout relieves this inhibition, upregulates CD69, CD27, granzyme B, and IFN-γ, enhancing cytotoxicity	Enhances CD8^+^ T cell effector function	Cell + Animal	(29)
DDX3Y (DBY)	HSCT	Transplant immunity	Half of male patients receiving female donor grafts develop IgG antibodies against recombinant DBY protein and DBY-specific CD4^+^ T cells	Induces alloantibody and CD4^+^ responses	Clinical	(41)
DDX3Y (DBY)	Kidney transplantation	Transplant immunity	Female recipients of male kidney grafts show a propensity for *de novo* anti-DDX3Y antibody development post-transplant, which is significantly positively correlated with acute rejection	Associated with acute rejection	Clinical	(42)
DDX3Y (DBY)	Skin transplantation	Transplant immunity	Antagonizing TCR contact sites of DBY expands peripheral CD4^+^CD25^+^ T cells, upregulates Foxp3 mRNA, and induces partial clonal deletion of effector T cells, promoting graft tolerance	Promotes Treg and induces tolerance	Animal	(43)
DDX39B	Multiple sclerosis	Autoimmunity	DDX39B knockdown reduces FOXP3 expression in CD4^+^CD25^+^ Tregs, causing loss of immunosuppressive features	Attenuates Treg suppressive function	Cell	(36)
DHX9	Multiple sclerosis	Autoimmunity	DHX9 is upregulated in CD4^+^ T cells from EAE mice and MS patients; T cell-specific Dhx9 knockout significantly reduces Th17 differentiation and ameliorates EAE symptoms	Promotes Th17 differentiation	Animal	(25)
DDX21	Inflammatory bowel disease	Autoimmunity	Ensures ribosome biogenesis upon T cell activation and promotes TFDP1 mRNA translation, regulating CD4^+^ T cell proliferation	Promotes CD4^+^ T cell proliferation	Animal + Cell	(24)
DDX5	Inflammatory bowel disease	Autoimmunity	Restricts HIF1α and IL-10 expression in RORγt^+^ Tregs	Restricts Treg suppression	Animal + Cell	(31)
DHX9	Rheumatoid arthritis	Autoimmunity	Enhances chromatin accessibility via its helicase activity, promoting SMAD2/3, STAT3, and IRF4 binding to the Il17a and Rorc loci to drive Th17 differentiation; synergizes with Nono to regulate Il17a promoter transcription, forming a positive feedback loop that amplifies Th17 differentiation	Promotes Th17 differentiation	Animal + Clinical	(25)
DDX5	Systemic lupus erythematosus	Autoimmunity	Serves as a novel serological marker for SLE; anti-DDX5 monoclonal antibody used for SLE diagnosis and differential diagnosis	Serological diagnostic marker	Clinical	(39)
DDX6	Sjögren’s syndrome	Autoimmunity	DDX6-CXCR5 risk locus SNPs (rs57494551, rs4936443, rs4938572, rs7117261, rs4938573) coordinately regulate multiple immune-related genes (DDX6, CXCR5, PHLDB1) via long-range chromatin loops; rs4938573 reduces CXCR5 promoter activity and downregulates CXCR5, a key migratory molecule for Tfh cells	Affects Tfh migration and immune gene expression	Genetic association + Cell	(40)
DHX9	Lymphocytic choriomeningitis virus	Anti-infection immunity	Binds LCK via its DSRM domain, ensuring CD3ϵ-LCK-ZAP70 signal axis transduction, promoting ZAP70 phosphorylation and downstream ERK activation, critical for CD8^+^ T cell survival before the proliferative burst	Maintains CD8^+^ T cell survival	Animal	(16)
DHX9	COVID-19	Anti-infection immunity	DHX9 is highly expressed in effector CD8^+^ T cells from the peripheral blood of COVID-19 patients	Upregulated in effector CD8^+^ T cells	Clinical	(16)
DDX5	Lymphocytic choriomeningitis virus	Anti-infection immunity	Suppresses expression of TCF1 and Eomes, key transcription factors for TCM differentiation and stem-like characteristics, driving CD8^+^ T cell differentiation toward the effector lineage	Drives effector differentiation	Animal	(34)

## 7 Translational applications

### 7.1 Analytical feasibility

Multiple DExD/H-box helicase members are amenable to detection in clinical specimens using established methodologies. Quantitative PCR and ELISA enable measurement of mRNA and protein levels for helicases such as DHX9 and DDX3X, with an absolute quantification system for DHX9 demonstrating robust linearity and reproducibility (53). The Olink high-throughput platform allows simultaneous quantitation of multiple immune-related proteins in minimal serum volumes and has successfully identified differentially expressed proteins including DDX58 (54). Additionally, exosomes derived from urine or plasma can be enriched via ultracentrifugation and subjected to Western blot or microarray analysis for integrins or circular RNAs. These approaches cover multi-level detection from gene to protein and from tissues to body fluids, yet cross-laboratory standardization and sample comparability require further optimization.

### 7.2 Therapeutic potential

Accumulating evidence indicates that several DExD/H-box helicases critically regulate T-cell differentiation, function, and fate determination, offering potential molecular targets for T-cell-directed immunomodulation. DDX3X participates in T-cell exhaustion and FOXP3 splicing regulation; its inhibitor RK-33 reverses CD4^+^ and CD8^+^ T-cell loss and ameliorates immunopathology in sepsis models (52, 55), while DDX3X inhibition also enhances intratumoral CD8^+^ T-cell infiltration, suggesting combinatorial potential in cancer immunotherapy (55). DDX39B serves as a critical activator of FOXP3 pre-mRNA splicing, and its expression or functional status directly influences Treg suppressive capacity; enhancing DDX39B activity may stabilize the Treg lineage for therapeutic benefit in autoimmune diseases (17). DDX5 regulates CD8^+^ T-cell memory differentiation, and its deficiency promotes central memory T-cell formation, indicating that DDX5 inhibitors might augment the persistence of immunotherapy by expanding TCF1^+^ stem-like CD8^+^ T cells (34). These findings provide a rationale for modulating T-cell functional states through targeted helicase intervention; however, most evidence derives from murine models or *in vitro* studies, and validation in primary human cells along with the feasibility of targeting strategies warrant further assessment.

### 7.3 Clinical relevance

In recent years, multiple DDX/DHX family members show potential clinical relevance, with potential applications in tumor prognosis, immunotherapy response, and autoimmune disease monitoring. In cancer immunotherapy, DDX3Y and USP9Y expression in circulating immune cells distinguishes PD-1/PD-L1 responders from non-responders, with high expression correlating with prolonged progression-free survival (56). In relapsed/refractory DLBCL, DDX3X mutations predict poor survival following CAR-T cell therapy (57); in NK/T-cell lymphoma, the mutation frequency is approximately 20% and associates with adverse outcomes (58). DLBCL patients with DDX3X mutations have a 5-year OS of only 22%, versus 72% in wild-type patients (59). DDX5, via the NAT10/ac4C/DDX5/HMGB1 axis in nasopharyngeal carcinoma, suppresses CD4^+^ and CD8^+^ T-cell function and promotes tumor progression (60); pan-cancer analyses show DDX5 expression correlates with CD8^+^ T-cell infiltration and predicts poor OS across multiple cancers (61). In sepsis, DDX3X mRNA levels in T cells correlate with disease severity and mortality (51). Most findings, however, stem from retrospective or small exploratory studies and require prospective validation.

## 8 Future perspectives

DExD/H-box RNA helicases serve as multifaceted regulators of T cell immunity across multiple biological processes. They actively participate in TCR signal transduction, modulate RNA splicing-dependent Treg lineage programming, and function as upstream pattern recognition sensors in antigen-presenting cells. As critical epigenetic regulators, these helicases also shape effector differentiation and memory cell generation in CD8^+^ T cells. By virtue of their RNA-binding and unwinding capacity, this protein family links innate immune detection to adaptive T cell responses, establishing a molecular axis that integrates cellular RNA metabolism with immune homeostasis and functional regulation.

Notwithstanding these emerging findings, several unresolved challenges continue to restrict mechanistic depth and translational progress. A large proportion of DExD/H-box family members remain poorly characterized in T cell contexts. Current mechanistic evidence is predominantly derived from murine models and transformed cell lines, with scarce systematic validation in primary human T cells. This experimental gap makes it difficult to distinguish bona fide immunoregulatory functions from artificial outcomes induced by species-specific differences and experimental contexts. The inherent functional pleiotropy of individual helicases further complicates targeted therapeutic development, as distinct and even opposing biological effects are frequently observed across different T cell subpopulations and disease scenarios. Such context-dependent outcomes are shaped by lineage and differentiation stage, cytokine and signaling milieu, selective use of catalytic versus non-catalytic domains, partner proteins and lncRNAs, post-translational modifications, subcellular localization, and the availability of specific RNA or chromatin substrates. Meanwhile, functional redundancy and compensatory crosstalk among family members still remain underappreciated, hindering accurate interpretation of single-gene intervention results.

Future research endeavors should focus on comprehensive and context-dependent characterization of helicase biology throughout T cell development, activation, metabolic adaptation, and long-term memory maintenance. In-depth mechanistic work is also required to unpack their non-canonical activities as signaling adaptors, epigenetic modulators, and metabolic sensors, with particular attention to how post-translational modifications and protein interactomes reshape their functional versatility. From a translational perspective, structural insights into individual helicase members will facilitate the development of selective targeting strategies. Subsequent clinical validation in patient cohorts and combinatorial therapeutic design with immune checkpoint blockade, JAK inhibition, or conventional immunosuppressive regimens will be important for translating the potential of this helicase family into clinical applications in immunomodulatory therapy.
